# Cell death-based approaches in treatment of the urinary tract-associated diseases: a fight for survival in the killing fields

**DOI:** 10.1038/s41419-017-0043-2

**Published:** 2018-01-25

**Authors:** Diego Martin-Sanchez, Miguel Fontecha-Barriuso, Maria Dolores Sanchez-Niño, Adrian M. Ramos, Ramiro Cabello, Carmen Gonzalez-Enguita, Andreas Linkermann, Ana Belén Sanz, Alberto Ortiz

**Affiliations:** 1 Research Institute-Fundacion Jimenez Diaz, Autonoma University, Madrid, Spain; 2 IRSIN, Madrid, Spain; 3 REDINREN, Madrid, Spain; 4 Department of Internal Medicine III, Division of Nephrology, University Hospital Carl Gustav Carus at the Technische Universität Dresden, Dresden, Germany

## Abstract

Urinary tract-associated diseases comprise a complex set of disorders with a variety of etiologic agents and therapeutic approaches and a huge global burden of disease, estimated at around 1 million deaths per year. These diseases include cancer (mainly prostate, renal, and bladder), urinary tract infections, and urolithiasis. Cell death plays a key role in the pathogenesis and therapy of these conditions. During urinary tract infections, invading bacteria may either promote or prevent host cell death by interfering with cell death pathways. This has been studied in detail for uropathogenic *E. coli* (UPEC). Inhibition of host cell death may allow intracellular persistence of live bacteria, while promoting host cell death causes tissue damage and releases the microbes. Both crystals and urinary tract obstruction lead to tubular cell death and kidney injury. Among the pathomechanisms, apoptosis, necroptosis, and autophagy represent key processes. With respect to malignant disorders, traditional therapeutic efforts have focused on directly promoting cancer cell death. This may exploit tumor-specific characteristics, such as targeting Vascular Endothelial Growth Factor (VEGF) signaling and mammalian Target of Rapamycin (mTOR) activity in renal cancer and inducing survival factor deprivation by targeting androgen signaling in prostate cancer. An area of intense research is the use of immune checkpoint inhibitors, aiming at unleashing the full potential of immune cells to kill cancer cells. In the future, this may be combined with additional approaches exploiting intrinsic sensitivities to specific modes of cell death such as necroptosis and ferroptosis. Here, we review the contribution of diverse cell death mechanisms to the pathogenesis of urinary tract-associated diseases as well as the potential for novel therapeutic approaches based on an improved molecular understanding of these mechanisms.

## Facts

- Cell death plays a key role in the pathogenesis and therapy of urological conditions such as cancer (prostate, renal, and bladder), urinary tract infections, crystalluria, and urinary tract obstruction.
- Uropathogenic *E. coli* (UPEC) invade urinary tract epithelial cells and leukocytes and may either promote or prevent host cell death by interfering with cell death pathways.
- Both crystals and urinary tract obstruction lead to tubular cell death and kidney injury.
- Urinary tract tumors develop resistance to apoptosis through diverse mechanisms, including Von-Hippel Lindau (VHL) mutations in clear cell kidney cancer and resistance to survival factor deprivation in prostate cancer.
- However, urinary tract tumors may be more sensitive to programmed necrosis, including necroptosis and ferroptosis.
- In addition, urinary tract tumors may promote death or exhaustion of antitumor immune cells. This is now targeted clinically with immune checkpoint inhibitors.

## Open questions

- How should uropathogenic *E. coli* (UPEC) modulation of host cell death be targeted to optimize bacterial clearance while limiting infection-associated tissue injury?
- How can a detailed knowledge of molecular mechanisms that allow urinary tract cancer to escape apoptosis be modulated to enhance tumor cell death?
- How will the improved understanding of tumor cell sensitivity to necroptosis and ferroptosis be translated to novel approaches to treat urinary tract cancer?
- Can induction of tumor cell necroptosis and ferroptosis be used to enhance the antitumor immune response?
- Is there a role for the therapeutic manipulation of NETosis in urinary tract disease?

## The burden of urinary tract diseases

Urinary tract diseases comprise a complex set of disorders with a variety of etiologic agents and therapeutic approaches. According to the Global Burden of Disease study, prostate cancer is the urinary tract disease with the heaviest worldwide burden. In 2015, it accounted for 366,000 deaths and 1,150,000 years lived-with-disability (YLD), and is followed by urinary tract infection (UTI, 196,000 deaths) and bladder and kidney cancer (188,000 and 137,000 deaths, respectively)^1,2^. In terms of YLDs, the burden of bladder and renal cancer (267,000 and 202,000, respectively) is higher than UTI and urolithiasis (100,000 and 90,000, respectively). Overall, both deaths and YLDs due to urinary tract conditions increased around 30% from 1995 to 2015, although the increase was 60% for prostate cancer YLDs^1,2^. The Supplementary Appendix summarizes current management of urinary tract disease.

The role of cell death in urinary tract disease is complex. Tumor cells have developed tools to enhance their own survival and to promote death or exhaustion of immune cells, while immune cells have tools to kill cancer cells and bacteria. Bacteria manipulate the host cell death mechanisms, increasing or decreasing cell survival, depending on bacterial strain, target host cell, and context. An improved understanding of the molecular mediators underlying the fight for survival in these killing fields will help optimize the therapeutic approach to diverse urinary tract conditions, aiming at preserving parenchymal cell and leukocyte viability while maximizing bacterial death in UTI, preserving parenchymal cell survival in urinary tract obstruction, and promoting tumor cell death while limiting death of antitumor leukocytes. We review the contribution of diverse cell death mechanisms to the pathogenesis of urinary tract-associated diseases and potential for novel therapeutic approaches based on an improved understanding of these mechanisms.

## Cell death mechanisms

Cell death is classified by morphological and mainly by biochemical and functional features into apoptosis or necrosis^3^.

Apoptosis can be executed through intrinsic or extrinsic pathways^4^. The extrinsic pathway is triggered by ligation of death receptors by tumor necrosis factor superfamily (TNFSF) members, such as TNF, Fas ligand (FasL), TNF-related apoptosis-inducing ligand (TRAIL), and TNF-like weak inducer of apoptosis (TWEAK), leading to activation of caspase-8^5^. The intrinsic pathway is initiated by cell stress causing outer mitochondrial membrane permeabilization and release of cytochrome c, which forms the apoptosome with Apaf-1 to activate caspase-9. Both, caspase-8 or caspase-9, activate executioner caspases, such as caspase-3. Several proteins positively and negatively regulate caspase activation (Supplementary Appendix).

Classically, necrosis has been considered as an accidental cell death pathway. However, different subroutines of regulated necrosis such as necroptosis, ferroptosis, mitochondria permeability transition-regulated necrosis, pyroptosis, and NETosis are of potential therapeutic interest^6,7^. During necrosis, the plasma membrane is disrupted, and release of intracellular contents triggers inflammation and immunogenic responses^8^.

Necroptosis is the best characterized form of regulated necrosis. Necroptosis requires the interaction of receptor-interacting protein serine/threonine kinase 1 (RIPK1) and RIPK3 and mixed lineage kinase domain-like protein (MLKL) phosphorylation. Activation of cell death receptors leads to RIPK1 activation and RIPK3 phosphorylation, and then phospho-RIPK3 phosphorylates MLKL. MLKL phosphorylation results in plasma membrane translocation and subsequent recruitment of a yet not completely characterized complex machinery that promotes plasma membrane rupture. This machinery is regulated by membrane repair mechanisms and involves the ESCRT-III complex^9^. Necroptosis can be modulated therapeutically with RIPK1 inhibitors (e.g., necrostatin-1s, ponatinib)^10^. Ferroptosis is characterized by accumulation of lipid peroxidation products resulting from dysfunction of glutathione peroxidase 4 (GPX4) and requires free cellular iron. GPX4 is a selenoprotein, which uses reduces glutathione to catalyze the reduction of hydrogen peroxide, protecting cells against damage by lipid peroxidation^11^. Erastin and RSL3 are inducers of ferroptosis. RSL3 directly inhibits GPX4^11^. Erastin inhibits the antiporter system Xc-, reducing the import into cells of cystine, a precursor of glutathione^12^. Ferroptosis and necroptosis of parenchymal cells may coexist in the same tissue. The pathophysiological relevance of necroptosis has been demonstrated in diverse preclinical models of disease, including acute kidney injury^13–18^ as recently reviewed^19^. Ferroptosis also contributes to injury to the kidney and other organs^20–23^. However, definitive proof of their role in human disease requires interventional studies specifically targeting these cell death pathways, which have not been performed yet.

Pyroptosis and NETosis occur in leukocytes. Pyroptosis is a highly inflammatory cell death predominantly occurring in macrophages and dendritic cells^7,24^. Contrary to other necrosis pathways, pyroptosis is caspase-dependent. Activation of macrophages or dendritic cells by danger-associated molecular patterns (DAMPs) or pathogen-associated molecular patterns (PAMPs) promotes the activation of the NOD-like receptor protein 3 (NLRP3) inflammasome that activates caspase-1 and caspase-11, and these caspases process pro-interleukin (IL)-18 and pro-IL-1β to mature proinflammatory cytokines. IL-18 and IL-1β accumulate in the intracellular compartment, but ultimately are released to the extracellular milieu when plasma membrane integrity is lost. The caspase target gasdermin-D mediates rupture of the plasma membrane^25^. PAMP activation of inflammasomes represents a host defense mechanism against bacterial infection, but DAMPs can activate sterile pathological inflammation^26^.

NETosis is a form of regulated necrosis typically observed in neutrophils. NETosis is mainly activated as an antibacterial immune defense mechanism, although it can be also activated by sterile stimuli such as cytokines, immune complexes, or autoantibodies^27,28^. During NETosis, neutrophils release neutrophil extracellular traps (NETs) composed of chromatin and histones, which immobilize and kill bacteria. However, aberrant NETosis may trigger autoimmune disorders such as lupus erythematosus, vasculitis, or rheumatoid arthritis^27^. The formation of NETs by activated neutrophils requires NADPH-oxidase-mediated reactive oxygen species (ROS) production and autophagy. Morphologically, NETosis is characterized by disintegration of the nuclear envelope and most granule membranes, massive vacuolization, and decondensation of nuclear chromatin^29^.

## Cell death in UTI

**Fig. 1:**
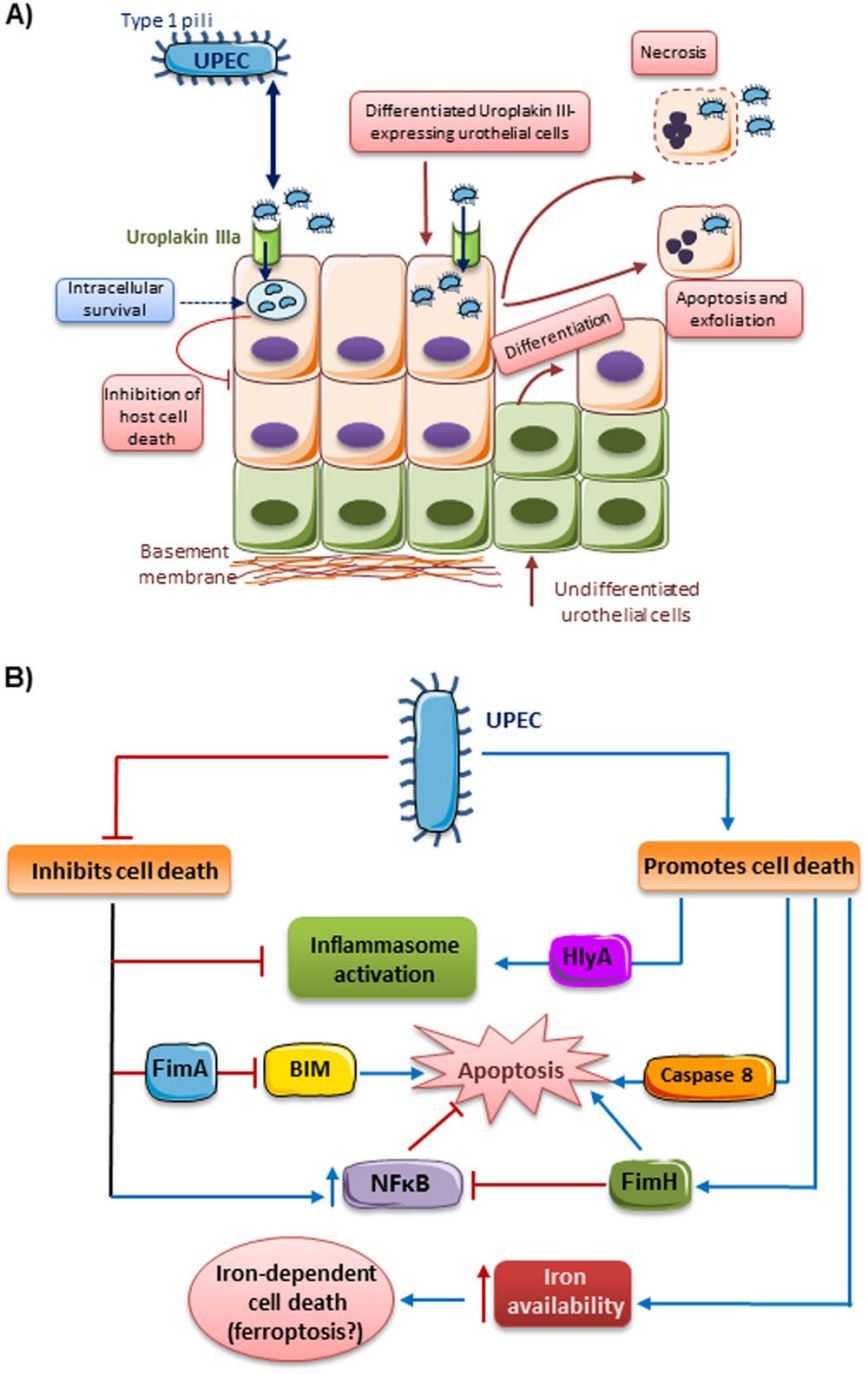
Cell death and survival during UTI. Factors modulating urothelial cell survival are summarized, but UPEC also interferes with cell death and survival in leukocytes, tubular epithelial cells, and others. **a** UPEC type 1 pili binds to uroplakin IIIa expressed in differentiated urothelial cells, allowing bacterial access to urothelial cells. This may trigger two different responses, depending on bacterial strain, stage of the infection, and the host cell context: (a) urothelial cell apoptosis or necrosis leading to tissue injury and shedding of injured cells and bacteria or (b) inhibition of host cell death favoring the survival of intracellular live bacteria. **b** Bacteria modulate cell survival through different mechanisms that may be strain-specific. UPEC may both promote (if HlyA is expressed) or inhibit inflammasome activation-dependent cell death. They may also promote an iron-dependent cell death that has not yet been characterized as ferroptosis

The interactions of UPEC with genitourinary epithelium and leukocytes have been studied in most detail^30^ (Fig. 1). However, findings may not apply to other bacteria, which may have their own strategies to escape killing by innate immunity defenses^31^. UPEC manipulates cell death mechanisms either to suppress or promote cell death in epithelial cells or leukocytes, as described in detail in the Supplementary Appendix^30^. While this may seem contradictory, the net effect will depend on the specific UPEC strain, stage of the infection, and target cell microenvironment. Death of host cells helps to get rid of infected cells and their contents of intracellular live bacteria, but promotes tissue in jury and pathogen release. Depending on the magnitude of live bacteria release, stage of the infection, and efficacy of antibiotics or extracellular antibacterial defenses, release of live bacteria may result in killing of bacteria in the extracellular space, infection of adjacent cells, recurrent infection, or triggering potentially more severe upper UTI^30^. By contrast, inhibition of host cell death may decrease tissue injury but facilitate pathogen persistence inside the cells. The fact that both promotion and prevention of host cell death by therapeutic intervention has both potentially beneficial and detrimental aspects poses a problem from the point of view of the design of therapeutic strategies.

## Cell death, crystalluria, and urolithiasis

Several crystals of clinical relevance may cause urinary tract cell death, although most studies have focused on renal tubular epithelial cells. Oxalic acid has been studied in most detail. Primary (genetic) or secondary hyperoxaluria may cause crystalluria, urolithiasis, and kidney injury. The interaction of oxalate ions with renal epithelial cells may initiate programmed cell death, either apoptosis or necrosis, as detailed in the Supplementary Appendix^32,33^.

## Cell death in urinary tract obstruction

Urinary tract obstruction is a frequent phenomenon in the clinic than is usually corrected by timely surgery or intervention. In addition, it has extensively been used as a preclinical model of accelerated chronic kidney disease, as detailed in the Supplementary Appendix^34^. Inflammation and low levels of pro-survival factors are likely drivers of tubular cell apoptosis since deficiency of inflammatory cytokines or administration of pro-survival cytokines decreased tubular cell apoptosis^35,36^.

## Cell death and genitourinary cancer

The three main types of genitourinary cancer have specific features regarding their sensitivity to diverse forms of cell death (Table 1), which result in sensitization or resistance to specific forms of cell death and can be exploited therapeutically (Table 2).

**Table 1:** Molecular mechanisms for evasion from apoptosis in urinary tract cancer. Selected examples

**Fig. 2:**
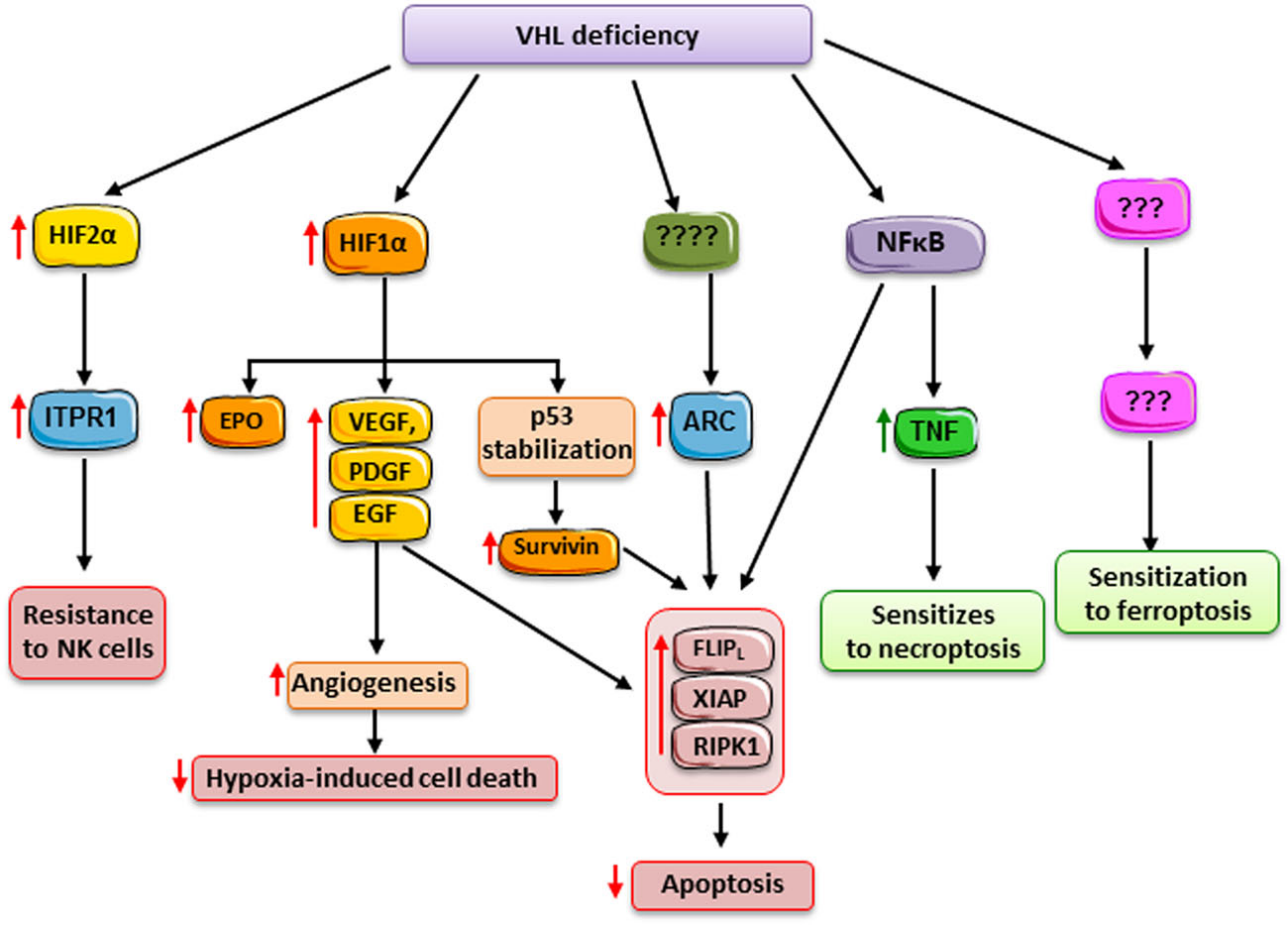
VHL mutations and RCC resistance to cell death. VHL is frequently mutated in hereditary and spontaneous RCC. This modifies the sensitivity to cell death through different pathways. Activation of HIF-1α and HIF-2α promotes the expression of intracellular and extracellular survival factors and of factors that promote angiogenesis and oxygen delivery, thus protecting from NK toxicity, hypoxia, and apoptosis. NFκB activation also protects from apoptosis, but creates an inflammatory milieu that sensitizes to necroptosis. The molecular basis for the increased sensitivity to ferroptosis remains currently unexplained

*Kidney cancer*
VHL deficiency (Fig. 2)
Mutations in astrocyte elevated gene-1 (AEG-1) or zinc-finger protein X-linked (ZFX)
Autophagy and mTOR activation
*Urothelial cancer*
Autophagy and increased expression of Beclin-1 and Atg7
Downregulation of cell surface Fas and release of soluble Fas
Caspase-3 downregulation
Bcl2 and survivin upregulation
*Prostate cancer*
Increased Bcl2 expression
BAD phosphorylation
Glucocorticoid receptor activation
Fn14 downregulation

**Table 2:** Selected examples of mutations or gene expression modifications in urinary tract cancer that modulate the sensitivity to cell death triggers as compared to nonrenal cells

Tumor	Cell survival-related changes	Cell death trigger	Consequences	Reference
RCC				
Mutated VHL	Increased HIF increased VEGF and survival factors, survival factors' sensitivity	Hypoxia or survival factors deprivation-induced apoptosis	Resistance	^39,110–112^
	NFκB activation leading to ↑TNFα, ↑RIPK1, and ↑RIPK3	Inflammatory cytokine-induced apoptosis	Resistance	^42,43^
		NFκB inhibitor-induced necroptosis	Sensitization	^45^
		Inflammatory cytokines triggered necroptosis	Sensitization	^46^
		Cystine deprivation-triggered ferroptosis. Ferroptosis-inducing drugs	Sensitization	^11,47,49^
Mutation in anti-apoptotic proteins	mTOR activation and autophagy	Apoptosis	Resistance	^53,54^
		mTOR inhibitors or autophagy inhibitor-induced necroptosis	Sensitization	
	PD-L1, B7-H4 expression	T-cell-induced cell death	Resistance through T-cell exhaustion	^55,56^
	CD70 expression	T-cell-induced cell death	Resistance trough promotion of K cell death	^57,58^
Bladder cancer	Induced expression of autophagy-related proteins	Autophagy inhibitors triggered apoptosis	Sensitization	^59–61^
	Release of soluble TNF superfamily receptors	TNF superfamily-induced apoptosis	Resistance	^62,63^
Prostate cancer	Initial stage: dependence of androgen as survival factors	Survival factors triggered apoptosis	Sensitization	^70,74^
	Advanced stage: increased expression of anti-apoptotic protein Bcl2 family members, decreased activity of proapoptotic Bcl2 family members	Survival factors triggered apoptosis	Resistance	^71–73^
	Decreased expression of TNF receptors superfamily members	TNF superfamily-induced apoptosis	Resistance	^75–78^

### Kidney cancer

Inherited RCC syndromes and around 70% of non-familial RCC have mutations in the Von-Hippel Lindau (VHL) gene encoding the VHL protein (pVHL)^37,38^. This may confer both resistance to apoptosis and sensitivity to necroptosis and ferroptosis upon the presence of adequate triggers (Fig. 2). pVHL is part of an E3–ubiquitin ligase complex targeting proteins for proteasomal degradation and interacts with more than 30 binding partners. Mutations in VHL provide resistance to cell death through a variety of molecular mechanisms. The best characterized is prevention of hypoxia-inducible factor (HIF) degradation, leading to a cell response that simulates the response to hypoxia that protects the tumor from hypoxia-induced death. The key HIF protein appears to be HIF-2, since in VHL-reconstituted cells, constitutive HIF-2 activity restores tumorigenesis^39^. HIF-responsive genes and HIF-dependent pathways include Vascular Endothelial Growth Factorm and are summarized in the Supplementary Appendix.

A well-characterized HIF-independent consequence of VHL deficiency is NF-κB activation. As previously indicated, NF-κB drives the transcription of pro-survival factors, such as XIAP or cellular FLICE inhibitory protein-long (c-FLIP_L_), and also of inflammatory cytokines such as TNF^40–43^. This pre-existing high levels of TNFα render VHL-deficient cells susceptible to necrosis triggered by cystine deprivation through a TNFα–RIPK1/RIPK3–MLKL pathway, since they rely on intact RIPK1 to inhibit TNF-induced apoptosis^44^. NFκB activation by interferon gamma (IFNγ) promotes the expression of pro-survival genes in RCC cells, but an inhibitor of NFκB switches the response to IFNγ from survival to necroptosis^45^. High-grade tumors express higher levels of necroptotic proteins RIPK1 and RIPK3 than normal tubular cells or low-grade tumors, and necroptosis may be the dominant pathway of cell death induced by TNF^46^. This observation offers the opportunity to design strategies to bypass the apoptosis resistance of VHL-deficient RCC cells. In addition, the high sensitivity to cystine deprivation may contribute to the exquisite sensitivity of RCC cells to erastin-induced ferroptosis^11,47^. Knockdown of GPX4 using short interfering RNAs was sufficient to kill RCC cell lines, via a characteristic ferroptotic death^11^. Indeed, erastin and other ferroptosis-inducing compounds, such piperazine erastin, and Ras-Selective RSL3, prevented tumor growth in a xenograft model^11,20^, providing a possible therapeutic application. In this regard, sorafenib, a tyrosine kinase inhibitor used to treat RCC^48^, is also a ferroptosis inducer, indirectly supporting the clinical relevance of ferroptosis in RCCs^49^. Importantly, sorafenib also modulates the necroptosis machinery^50^. Additional oncogenes mutated in RCC or tumor-associated genes also promote resistance to apoptosis. Thus, knockdown of astrocyte elevated gene-1 or zinc-finger protein X-linked leads to activation of Bax and caspase-3, and apoptosis in RCC^51,52^.

Autophagy and mammalian Target of Rapamycin activation are survival mechanisms in RCC. Indeed, mammalian Target of Rapamycin inhibitors are part to the current therapeutic armamentarium against RCC^53^. Inhibition of autophagy and mammalian Target of Rapamycin promotes necroptosis, to which RCC cells may be sensitized, as indicated above^54^.

Finally, RCC fights back the immune system by expressing molecules such as programmed death ligand 1 (PD-L1, B7-H1) and B7-H4 that interfere with antitumor defenses by activating T-cell receptors that limit T-cell responses and prevent autoimmunity, such as the immune checkpoint inhibitor receptor programmed cell death protein 1^55,56^. In addition, RCC may express TNFSF cytokines that may directly kill lymphocytes, such as CD70 and FasL^57^. In this regard, stimulation of the immune system with IFNα and high-dose IL-2 has been used to treat RCC^58^.

### Urothelial cancer

The mechanisms that trigger cell death in urothelial cancers are poorly understood. Indeed, currently available chemotherapeutic agents directly killing urothelial cancer cells have limited efficacy. Autophagy may contribute to treatment resistance in urothelial cancer and targeting autophagy may overcome resistance^59^. Autophagic vesicles and levels of autophagy proteins, such as Beclin-1 and Atg7, were increased in human urothelial cancer^60^. Chemical inhibition of autophagy resulted in activation of caspase-9 and apoptosis in cultured urothelial cancer cell lines^60,61^.

Bladder cancer cells downregulate cell surface lethal receptor Fas and release soluble Fas, which behaves as a decoy receptor for FasL^62,63^. Indeed, urinary soluble Fas was a predictor of bladder cancer^62^, while membrane Fas downregulation was associated with a more advanced stage and poorer prognosis^64^. Moreover, assessment of apoptosis markers provides prognostic information in bladder cancer. Specifically, the combination of caspase-3 downregulation, and Bcl2, p53, and survivin upregulation, was associated with higher pathological stage and worse prognosis^65–69^. However, there are no studies addressing regulated necrosis dysregulation in urothelial cancer.

### Prostate cancer

Cells are classified according to their dependence on the androgen receptor (AR). In androgen-dependent prostate cancer cells, AR depletion or anti-androgen therapy induce features of survival factor deprivation, such as cell cycle arrest and apoptosis^70^. However, following initial regression, tumors often return in an AR-independent form, which is frequently lethal.

Resistance to apoptosis has been observed in prostate cancer cells resistant to androgen depletion. In prostate cancer xenografts, castration-resistant cells expressed the anti-apoptotic protein Bcl2^71^ and high Bcl2 staining was found in biopsies of patients with metastasis^72^. Phosphorylation of the pro-apoptotic protein BAD by PI3K protects prostate cancer cells from apoptosis. Moreover, the anti-apoptotic protein Mcl-1 promotes survival, even if BAD is phosphorylated^73^. A recent study suggests that the glucocorticoid receptor may replace AR to mediate prostate cancer survival, but the impact on cell death has not been addressed^74^.

TNFSF members can promote prostate cancer cell death. AR-independent PC3 cells express the TWEAK receptor Fn14 and are sensitive to TWEAK-induced apoptosis, while AR-dependent LnCAP cells do not express Fn14 and are TWEAK-resistant^75^. Expression of TRAIL and its lethal receptor TRAIL-R2 was higher and TRAIL decoy receptor lower in androgen ablation-treated patients than in untreated patients^76,77^. In this sense, the flavonoid quercetin increased TRAIL-R2 expression enhancing TRAIL-mediated death in prostate cancer cells^78^. Thus, therapy that increases TNF receptor superfamily activation could contribute to initial tumor regression during androgen ablation therapy or even in androgen-resistant tumors through apoptosis induction.

### Cell death targeting in urinary tract-associated diseases

Therapeutic efforts to manipulate cell death in urinary tract-associated diseases may be aimed at decreasing cell death (e.g., tubular and endothelial cell death in urinary tract obstruction) or promoting cell death (e.g., cancer).

Despite preclinical advances (Table 3), there is yet no approach in clinical development or use to modulate cell death during UTI or to decrease cell death during urinary tract obstruction or crystalluria. During UTI, a delicate balance is present between the need to eliminate live foci of infection within cells and the resulting tissue damage. Ideally, a novel therapeutic strategy may both contribute to eliminate bacteria and preserve tissue integrity. In this regard, UPEC produces a pore-forming toxin and such toxins are thought to induce necroptosis of macrophages through activation of RIPK1, RIPK3, and MLKL by a mechanism involving loss of ion homeostasis at the plasma membrane, mitochondrial damage, ATP depletion, and the generation of ROS^79^. Blocking macrophage necroptosis induced by pore-forming toxin protected against *Serratia marcescens* hemorrhagic pneumonia, but the impact on UTI was not addressed. Thus, the impact of inhibitors of RIPK1, RIPK3, or MLKL should be explored for their effect on urinary tract bacterial burden and tissue injury, and pore-forming toxin blockers should be explored^79^. As long as effective antibiotics are available, the focus should be to prevent irreversible tissue injury. In this regard, there are differences between epithelial cells that depend on genitourinary tract location. Bladder urothelium has several layers and a high regenerative capacity from undifferentiated cells close to the basement membrane. As a result, dying cells are rapidly replaced, bladder urothelial cell death is unlikely to result in long-term sequelae, and antibiotics are usually rapidly effective. We do not envision intervention on cell death mechanisms unless bacterial strain resistant to all antibiotics develops. In this case, the therapeutic approach should be aimed at reducing the bacterial burden, even if this entails increasing bladder urothelium turnover. However, prevention of host cell death during epididymo-orchitis should be a therapeutic goal, since it may limit irreversible loss of somatic cells such as Sertoli cells and of germ cells, leading to infertility. Unfortunately, very little is known about the molecular mechanisms involved. A Pubmed search performed on 20 June 2017 found no reports of necroptosis or ferroptosis in the pathogenesis of UTI, although they are likely to play a role, given the caspase-independent forms of cell death that have been described and the requirement for iron in some forms of UPEC-triggered host cell death. Further characterization of the molecular mechanisms of cell death regarding the involvement of necroptosis or ferroptosis and their impact on bacterial clearance may expand the range of therapeutic tools available to intervene on cell death in UTI.

**Table 3:** Targets and pathways in preclinical development for diverse non-tumoral urinary tract conditions

Context	Key aim	Mechanism	Reference
Bladder infection			
Strong extracellular antibacterial defense	Prevent urothelial invasion (and death)	Block type 1 pili/uroplakin III interaction	^113–115^
	Eliminate bacteria	Increase urothelial cell death (e.g., favor HlyA activity)	
Weak antibacterial defense	Prevent urothelial cell death, promote inflammatory antibacterial defense	Prevent NFκB inactivation by UPEC metabolites and proteins	^116–118^
Orchiepididymitis	Prevent irreversible tissue injury	Decreases parenchymal cell death. Mechanism to be defined.	^119^
UTI, pore-forming toxin-containing bacteria	Prevent leukocyte death	Inhibit pore-forming toxin, inhibit macrophage necroptosis (e.g., RIPK1 inhibitor) prolonging neutrophil survival	^120–122^
Pyelonephritis	Prevent irreversible tissue injury	Decrease kidney tubular cell death (e.g., SKQR1 mitochondrial antioxidant)	^123^
Crystal-induced kidney injury	Prevent tissue injury	Decrease kidney tubular cell death (decrease necroptosis by targeting RIPK3 or MLKL, decrease NLRP3 inflammasome activation)	^124–126^
Urinary tract obstruction	Prevent irreversible tissue injury	Decrease kidney tubular cell death (decrease apoptosis by targeting inflammatory cytokines, dual targeting of Bax and Bak, targeting of Omi/HtrA2; potentially necrosis by targeting PARP1)	^127–129^

In contrast to the situation in non-malignant urinary tract diseases, cell death modulation is a cornerstone of anticancer therapy (Table 4). Therapeutic modulation of cell death in cancer has traditionally focused on finding agents that kill tumor cells, exploiting tumor cell vulnerabilities. More recently, efforts have been made to prevent tumor cells from killing or promoting exhaustion of innate and adaptive immunity cells, so as these leucocytes may accomplish their function of killing tumor cells^58,80,81^.

**Table 4:** Cell death targeting in urinary tract-associated cancer: approaches in clinical use or in clinical development aimed at increasing tumor cell death

Mutation-directed therapy. Kidney cell cancer^82^
VEGF signaling inhibitors (anti-VEGF or tyrosine kinase inhibitors)
mTOR inhibitors
Immune checkpoint inhibitors targeting the programmed death 1/programmed death ligand 1 (PD-1/PD-L1) or cytotoxic T-lymphocyte-associated protein 4 (CTLA-4) pathways: metastatic renal cell cancer or bladder cancer^80,129^. These drugs decrease T-cell exhaustion, thus enhancing their capacity to kill tumor cells
Survival factor-directed therapy. Anti-androgen therapy in advanced prostate cancer^92^
Cytoskeleton-targeted therapies (taxanes). Castration-resistant prostate cancer^93^
Additional approaches undergoing clinical trials
Sorafenib (tyrosine kinase inhibitor): castration-resistant prostate cancer^97,98^
Olaparib (PARP inhibitor): castration-resistant prostate cancer^102^
BET bromodomain protein inhibitors: cancer^103,130–157^

### Mutation-directed therapy

The current standard of therapy for RCC exploits knowledge about the consequences of VHL mutations. Thus, Vascular Endothelial Growth Factorm signaling inhibitors (anti-VEGF or tyrosine kinase inhibitors) and mammalian Target of Rapamycin inhibitors have proved efficacious and become the standard approach^82^. Despite the poor efficacy of IFN-γ monotherapy, the combination of IFN-γ with the proteasome inhibitor bortezomib killed RCCs by activating RIPK1-dependent necroptosis^45,83^. Bortezomib sensitizes otherwise resistant RCCs, at least in part, by inhibiting prosurvival NF-κB signaling, but the existence of additional mechanisms is almost certain^45,84^. RCC cells express high levels of necroptotic proteins RIPK1 and RIPK3, suggesting that they may be more sensitive to necroptosis^46^. mammalian Target of Rapamycin inhibition stimulates autophagy and eliminates RIPKs in RCCs, thus allowing scape from mammalian Target of Rapamycin inhibition. This is blocked by autophagy inhibition, which induces RIPK-dependent and ROS-dependent necroptosis in vitro and suppresses xenograft growth^54^. Thus, activating necroptosis or concomitant proteasome or autophagy inhibition may be avenues to increase RCC death.

Since mutated VHL signals through HIF, direct targeting of mutant VHL or of HIF has been considered. Indeed, restoration of functioning VHL restores many of the abnormalities of RCC cells with mutant alleles^85^. However, restoration of normal VHL would need a gene therapy approach. Direct targeting of HIF also has limitations. HIF activation is required for tissue and organism resistance to hypoxia, and, indeed, HIF activators have shown a beneficial cardiovascular risk profile (lower blood pressure, lower cholesterol) in clinical trials for uremic anemia^86^. However, a small molecule HIF-2 inhibitor (PT2399, PT2385) suppressed tumorigenesis in around 50% of human RCC lines and in a patient whose RCC was predicted to be sensitive to the drug. Mutations in HIF-2α and HIF-1β accounted for tumor resistance to the drug in some patients^39^.

### Immune checkpoint inhibitors

Immune-checkpoint inhibitors targeting the programmed death 1/programmed death ligand 1 (PD-1/PD-L1) or cytotoxic T-lymphocyte-associated protein 4 (CTLA-4) pathways are currently indicated to treat metastatic RCC or bladder cancer^80,81^. These include atezolizumab and nivolumab for bladder cancer and likely pembrolizumab in the near future, and nivolumab for RCC^81^. However, no beneficial effect of ipilimumab was observed in prostate cancer patients^87^. Cancer cell-associated PD-L1 increases apoptosis of antigen-specific human T-cell clones in culture and of activated tumor-reactive T cells in vivo, thus impairing antitumor defenses and promoting tumor growth^88^. In addition, PD-L1 renders tumor less susceptible to specific T-cell antigen receptor-mediated lysis by cytotoxic T cells^89^. However, the main effect of immune checkpoint inhibitors appears to be the rejuvenation of exhausted CD8 T cells^90^. T-cell exhaustion is a unique state characterized by loss of proliferative capacity and effector function that is driven by epigenetic modulation^90,91^.

### Survival factor-directed therapy

Androgen deprivation therapy with anti-androgens remains the main treatment approach for advanced prostate cancer and can suppress tumor growth for 12–24 months. However, it eventually fails and tumors progress into the castration-resistant stage^92^.

### Cytoskeleton-targeted therapies

The taxanes docetaxel and cabazitaxel are the current therapy for castration-resistant prostate cancer^93^. They target the cytoskeleton by stabilizing the interaction of β-tubulin subunits of microtubules, preventing depolymerization, and inducing G2M arrest and apoptosis^94^.

### Additional approaches

There is plethora of preclinical studies exploring compounds that may kill genitourinary cancer cells in culture. A detailed discussion is beyond the scope of the present review. However, we will mention several approaches to promote prostate cancer death using agents that are already in clinical use for other tumors or undergoing clinical trials.

Necroptosis induction could be a therapeutic option for AR-independent prostate cancer. The pro-mitotic protein PLK1 is upregulated in AR-independent prostate cancer cells, and PLK1 inhibitors promote necroptosis^95^. Moreover, the combination of simvastatin and metformin, two drugs in clinical use for hypercholesterolemia and diabetes, respectively, promotes necroptosis in metastatic AR-independent prostate cancer cells^96^.

Sorafenib is undergoing clinical trials for AR-resistant prostate cancer^97,98^. In AR-independent prostate cancer PC3 cells, sorafenib induces caspase-dependent cell death and this is potentiated by autophagy inhibition, whereas in AR-independent DU145 prostate cancer cells, it induces necroptosis and this is autophagy-dependent^99,100^. In addition, sorafenib is a ferroptosis inducer. However, the occurrence of ferroptosis in response to sorafenib in prostate cancer cells has not been studied.

Germline breast cancer 1 (BRCA1) and breast cancer 2 (BRCA2) gene mutations are implicated in prostate cancer predisposition and aggressiveness. Cancer cells with these mutations rely on PARP to repair DNA and divide^101^. Olaparib is an oral PARP inhibitor in clinical use for ovary cancer that blocks DNA repair and, when coupled with BRCA mutations, results in tumor cell death. In phase II clinical trials, including patients with advanced AR-independent prostate cancer, olaparib seemed efficacious and well tolerated^102^.

Several bromodomain and extraterminal domain (BET) protein inhibitors are undergoing clinical trials in cancer^103^. BET proteins are epigenetic modulators. The BET bromodomain inhibitor JQ1 induced apoptosis and downregulated AR-regulated gene transcription by preventing the interaction between the BET protein BRD4 and the AR in prostate cancer cells, thus reducing xenograft tumor volume in mice^104^.

## Perspectives

A more detailed understanding of cell death mechanisms during urinary tract disease may lead to designing novel therapeutic strategies, although the aims of these strategies may differ in different urinary tract diseases (Tables 3 and 4). Most clinically significant advances in the near future are expected in the field of urinary tract cancer. Tumors have incorporated mutations and changes in gene expression that allow them to evade a number of triggers of death (e.g., survival factor deprivation-induced apoptosis, hypoxia-induced apoptosis, killing by T cells, recruitment of autophagy to preserve cell survival). However, this very same adaptive mechanism may sensitize them to specific forms of cell death, by therapeutically interfering with the adaptive mechanisms or by triggering alternative forms of cell death (e.g., necroptosis, ferroptosis; Table 2), and this can be exploited therapeutically.

For RCC and bladder cancer, the very active area of research on immune checkpoint inhibitors has the potential to change the landscape of treatment. Research may involve not only optimizing the inhibition of the immune checkpoint, but also enhancing tumor cell death in response to innate and adaptive antitumor responses activated by this therapeutic approach. For RCC, these further developments may try to exploit the recently identified sensitivity of cells with VHL mutations to necroptosis and ferroptosis^11,44^ to develop novel antitumor strategies aimed at increasing RCC death. HIF-2 targeting has emerged as an alternative for some patients^39^. Novel therapeutic approaches may also take advantage of the growing field of epigenetic targeted therapies that is not limited to BET proteins^103^. As an example, miRNA-708 overexpression decreased the expression of c-FLIP_L_ and sensitized RCC to apoptosis^43^. miRNAs are already in clinical trials, although the potential for nephrotoxicity has been noted^105^. Further optimization of immune checkpoint inhibitor therapy is also expected for bladder cancer. Given the multiple mutations that characterize cancer, the availability of multiple agents that can be combined is the basic tool for a real personalized medicine approach.

AR-resistant prostate cancer is still problematic. A number of observations have described sensitivity of AR-resistant prostate cancer cells to death induced by certain triggers such as TWEAK, sorafenib, olaparib, and inhibition of BET proteins or PLK1, among others^75,95,99,102,104^. TWEAK activity modulation has already reached clinical development^106^, while inhibitors of BET proteins and genetic (TKM-080301) or small molecule (GSK461364, BI 2536) targeting of PLK1 are undergoing clinical trials in cancer^107^. Olaparib and sorafenib are undergoing clinical trials specifically in prostate cancer (www.clinicaltrials.gov; accessed 15 July 2017).

An issue that remains poorly explored is the role of neutrophil death or neutrophil-induced parenchymal or tumor cell death in urinary tract conditions. Neutrophils may release NETs when undergoing a specific form of cell death (NETosis) or release NETs while still alive (live NETosis)^108^. Although there are few studies in the specific urinary tract context, NETosis is thought to contribute to bacterial clearance but to have a deleterious role under conditions of sterile inflammation or cancer. In cancer, NETosis has been suggested to contribute to an increased thrombosis risk or to promote tumor growth and, thus, to be a therapeutic target^108^. However, neutrophils have long been thought to be key effectors of the innate immunity response against bladder urothelial tumors when local immunotherapy with Bacillus Calmette-Guérin (BCG) is applied^109^.

In conclusion, understanding the molecular mechanisms of cell death has led to significant advances in the treatment of genitourinary cancer and novel therapeutic approaches are under study that exploits recent advances in the field. Therapeutic approaches in clinical development aim at inactivating molecular mechanisms that help tumor cells withstand apoptosis triggered by survival factor deprivation, hypoxia, or lethal and proinflammatory cytokines, to prevent tumor cell-induced death or exhaustion of antitumor leukocytes and to exploit tumor cell sensitivity to necroptosis and ferroptosis, which sometimes is linked to the very same mechanisms that promote resistance to apoptosis. However, modulation of cell death has lagged behind in the clinic as a means to improve the outcomes of other urinary tract-associated diseases such as UTI, and urolithiasis and its consequences. In this regard, a number of preclinical studies have identified targets that would allow protecting parenchymal cells or antibacterial leukocytes while promoting bacterial clearance. These may range from prevention of bacterial entry (and induction of cell death) in urothelial cells; to promoting urothelial cell death and release of live intracellular bacteria when strong extracellular antibacterial defenses are likely to kill extracellular bacteria; and to promoting leukocyte survival by protecting them from bacteria-triggered forms of cell death.

## Electronic supplementary material

Supplementary table 1

Supplementary appendix
