# Supplementary material for: Cell death-based approaches in treatment of the urinary tract-associated diseases: a fight for survival in the killing fields
Source: Cell Death Dis. 2018 Jan 25;9(2):118. doi: 10.1038/s41419-017-0043-2 (PMC5833412; doi:10.1038/s41419-017-0043-2)
Supplement: Supplementary file 1 — Supplementary table 1 [file 41419_2017_43_MOESM1_ESM.docx]

**Supplementary table 1**.

| **Antibody** | **Target** |
| --- | --- |
| Atezolizumab | PD-L1 (Programmed death-ligand 1), CD274 |
| Nivolumab | PD1 (Programmed cell death 1), CD279 |
| Pembrolizumab | PD1 (Programmed cell death 1), CD279 |
| Ipilimumab | CTLA-4 (cytotoxic T-lymphocyte associated 4), CD152 |

| **Drug** | **Target** |
| --- | --- |
| Ponatinib | Tyrosine kinase inhibitor |
| Sorafenib | Tyrosine kinase inhibitor |
| Bortezomib | Proteasome inhibitor |
| Olaparib | PARP inhibitor |
