# Supplementary material for: Cell death-based approaches in treatment of the urinary tract-associated diseases: a fight for survival in the killing fields
Source: Cell Death Dis. 2018 Jan 25;9(2):118. doi: 10.1038/s41419-017-0043-2 (PMC5833412; doi:10.1038/s41419-017-0043-2)
Supplement: Supplementary file 2 — Supplementary appendix [file 41419_2017_43_MOESM2_ESM.docx]

**Supplementary appendix**

**Current management of urinary tract disease**

Prostate cancer remains the second most commonly diagnosed cancer in men, with an estimated 1.1 million diagnoses worldwide in 2012 ^1^. Therapy may consist of resection or survival factor (i.e. androgen) deprivation. However, tumors may eventually progress to a castration (androgen deprivation)-resistant stage that kills the patient. Bladder urothelial cancer and renal cell cancer (RCC) are treated by resection when possible. Chemotherapy based on cisplatin and gemcitabine for advanced bladder cancer was considered suboptimal. Several types of RCC are recognized, the most common being clear cell cancer, which is treated with vascular endothelial growth factor (VEGF)- and mammalian target of rapamycin (mTOR)-targeting drugs when advanced ^2^. Both advanced RCC and bladder cancer can now be treated with immune checkpoint inhibitors ^3, 4^.

UTI recurrence is a major issue ^5^. While acute cystitis is usually uncomplicated, pyelonephritis and urosepsis may be deadly. The most common pathogen is uropathogenic *Escherichia coli* (UPEC)^6^. Acute renal colic is the main clinical manifestation of urinary lithiasis. However, urolithiasis may also cause sepsis and urinary tract obstruction leading to hydronephrotic kidney atrophy ^7^. Crystalluria may cause kidney damage by itself or evolve to full blown urolithiasis.

**Molecular regulation of apoptosis**

The intrinsic and extrinsic pathways of apoptosis lead to caspase activation ^8^. There are positive and negative regulators of caspase activation. Antiapoptotic proteins include some B-cell lymphoma 2 (Bcl2) family members [Bcl2, B-cell lymphoma-extra-large (BclxL), myeloid cell leukemia 1 (Mcl-1)] and inhibitors of apoptosis proteins (IAPs), while pro-apoptotic proteins include some Bcl2 family members [Bcl-2-associated X (Bax), bcl-2 homologous antagonist/killer (Bak), Bcl-2-Interacting Mediator of cell death (BIM), Bcl-2-associated death promoter (BAD)], second mitochondria-derived activator of caspase (SMAC) and high-temperature requirement a2 (Omi/Htra2). Anti- and pro-apoptotic Bcl2 family proteins interact between themselves to determine activation of the mitochondrial pathway of apoptosis, while IAPs target caspases and are inhibited by SMAC or Omi/Htra2. Endoplasmic reticulum stress can also activate caspases and promote cell death ^9^. Apoptotic cells express “eat me” signals in the cell surface that are identified by receptors in macrophage and parenchymal cells ^10^. Eventually, apoptotic cells are engulfed by adjacent cells before loss of cell membrane permeability results in release of proinflammatory factors ^10, 11^. Apoptosis is implicated in numerous physiological and pathological biological processes, may be inconspicuous and is not considered pro-inflammatory as necroptosis is.

**Cell death in urinary tract infection (UTI)**

The interactions of UPEC with genitourinary epithelium and leukocytes have been studied in most detail ^12^ (**Figure 1**). However, findings may not apply to other bacteria, which may have their own strategies to escape killing by innate immunity defenses ^13^. UPEC manipulate cell death mechanisms either to suppress or promote cell death in epithelial cells or leukocytes ^12^. While this may seem contradictory, the net effect will depend on the specific UPEC strain, stage of the infection, and target cell microenvironment. Death of host cells helps to get rid of infected cells and their contents of intracellular live bacteria, but promotes tissue injury and pathogen release. Depending on the magnitude of live bacteria release, stage of the infection and efficacy of antibiotics or extracellular anti-bacterial defenses, release of live bacteria may result in killing of bacteria in the extracellular space, infection of adjacent cells, recurrent infection or triggering potentially more severe upper UTI ^12^. By contrast, inhibition of host cell death may decrease tissue injury but facilitate pathogen persistence inside the cells. The fact that both promotion and prevention of host cell death by therapeutic intervention has both potentially beneficial and detrimental aspects poses a problem from the point of view of the design of therapeutic strategies.

UPEC invade and persist within the urothelium by forming reservoirs within autophagosomes, avoiding degradation through neutralization of lysosomal pH ^12^. To favor intracellular persistence, UPEC may prevent urothelial cell death through several mechanisms, such as disruption of inflammasome activation or epigenetically silencing the expression of the proapoptotic protein BIM ^12, 14^. UPEC FimA monomers have been identified as inhibitors of apoptosis in infected host cells ^15^.

UPEC may also promote cell death in urothelium, tubular renal cells and leukocytes cells by mechanisms involving type 1 pili, toxins such as α-hemolysin (HlyA), modulation of iron availability, NF-κB blockade and others ^16, 17, 18, 19, 20, 21, 22, 23, 24, 25, 26^. Urothelial death may be considered both a defense mechanisms, preventing intracellular bacterial survival, and a maladaptive response, favoring the release of live bacteria, which may trigger recurrence of UTI if bacteria had been confined to intracellular reservoirs by the immune response. Its precise role in a specific individual and in the specific stage of infection will be context dependent. Type 1 pili are filamentous organelles mediating the attachment of UPEC to epithelial cells. Binding of the type 1 pilus adhesin FimH to uroplakin IIIa triggers intracellular signaling that promotes bacterial invasion and urothelial apoptosis involving caspase activation and host DNA fragmentation, with exfoliation of bacteria-laden superficial facet cells, followed by rapid reconstitution of the urothelium through differentiation of underlying cells ^16, 17, 18^. In this regard, there is a requirement for urothelial differentiation-associated uroplakin III expression for UPEC-induced apoptosis ^19^. FimH activates caspases via direct induction of the extrinsic pathway and indirect activation of the intrinsic pathway by caspase 8-mediated Bid cleavage ^20^. UPEC α-hemolysin (HlyA) induces caspase-1/caspase-4-dependent and NLRP3-dependent inflammatory urothelial death. Overexpression of HlyA during acute bladder infection induces more rapid and extensive exfoliation and reduces bladder bacterial burdens ^21^. In this case, cell death helps reduce bacterial burdens. Iron availability through ferritinophagy triggers bacterial over proliferation within autophagosomes and host cell death through iron-induced lysosomal damage. Host cell death is prevented by inhibiting autophagy or iron-regulatory proteins, or by iron chelation ^22^. However, ferroptosis inhibitors have not been specifically tested in this context. Additionally, UPEC may block NF-κB, a transcription factor that promotes the expression of anti-apoptotic and pro-inflammatory genes ^23^. NF-κB blockade results in promotion of apoptosis by pilus-independent mechanisms ^23^, as well as in decreased cytokine secretion and thus, suppression of the host innate immune response ^24^. In this case, eventual release of intracellular bacteria will be not be met by an effective antibacterial inflammatory response.

UPEC is not the only bacteria that modulate urothelial cell death. Additional non-uropathogenic bacteria may interact to trigger UTI through induction of urothelial cell death. In this regard, non-uropathogenic *G. vaginalis* is not an etiologic agent for UTI. However, *G. vaginalis* triggers urothelial cell apoptosis and exfoliation ^27^. This may lead to release of *E. coli* from latent bladder reservoirs and is thought to be, in the appropriate context, a cause of recurrent UTI ^27^.

UPEC toxins also induce caspase-independent cell death in renal proximal tubular cells ^28^. Renal cell death during pyelonephritis is dependent on mitochondrial oxidative stress. Thus, the mitochondria-targeted antioxidant Skulachev quinone R1 (SkQR1) decreased cell death and increased animal survival ^29^. Bacterial epididymo-orchitis is associated to an increase in TUNEL + cells of germ cells without caspase activation and in passive release of high mobility group protein B1 (HMGB1), suggestive of necrosis, localized to testes somatic cells, and associated to morphological evidence of damage to Seroli cells ^30^. Understanding the molecular mechanisms of cell death in this context may help reduce the incidence of male infertility secondary to irreversible tissue injury.

UPEC also modulate leukocyte death. UPEC activate an NLRP3-independent cell death in macrophages ^25^ and type 1 pili- and lipopolysaccharide-dependent apoptosis linked to oxidative stress in neutrophils while non-pathogenic *E. coli* delays neutrophil apoptosis via lipopolysaccharide activation of intracellular signaling ^26^. In this case, longer neutrophil survival helps in the control of bacterial loads and contributes to the non-uropathogenicity of the bacterial strain.

**Cell death, crystalluria and urolithiasis**

Several crystals of clinical relevance may cause urinary tract cell death, although most studies have focused on renal tubular epithelial cells. Oxalic acid has been studied in most detail. Primary (genetic) or secondary hyperoxaluria may cause crystalluria, urolithiasis and kidney injury. The interaction of oxalate ions with renal epithelial cells may initiate programmed cell death, either apoptosis or necrosis ^31, 32^. Crystal-induced cell death was defined as mostly apoptotic based on results from TUNEL assays, although the presence of cells with necrotic morphology was also noted in kidney tissue ^33^. However, TUNEL detects [DNA](https://en.wikipedia.org/wiki/DNA) fragmentation and is not as specific for apoptotic DNA fragmentation as initially thought, labeling damaged DNA in necrotic cells. Further studies disclosed that crystal size influences the type of cell death ^34^. Phagocytosis of calcium oxalate nanocrystals followed by their fusion with lysosomes may trigger apoptosis, but massive compromise and destruction of lysosomes may trigger necrosis. Nanocrystals can also induce apoptosis by directly damaging mitochondria, causing dissipation of the membrane potential, and by entering the nucleus and causing direct DNA cleavage. Micron-sized crystals may disrupt the plasma membrane and enter the cytoplasm, causing cell necrosis, DAMP release and lysosome swelling and destruction.

Necroptosis and the inflammasome appear to be instrumental in crystal-induced tubular cell death. Inhibition of necroptosis by targeting RIPK3 or MLKL protected tubular cells from calcium oxalate, monosodium urate, calcium pyrophosphate dihydrate and cystine crystals ^35^. Phagolysosome membranes destabilized by undigested crystals allow the release of cathepsin that further activates the RIPK1/RIPK3/MLKL pathway. In vivo, RIPK3 or MLKL deficiency prevents oxalate crystal-induced acute kidney injury ^36^. Inflammation, driven by DAMP release from necrotic cells and TLR ligation or by cytokine release, further amplified the occurrence of necroptosis. Pharmacological blockade or deficiency of NLRP3 also prevents renal injury induced by adenine or oxalate crystals ^37^. This involved prevention of inflammasome activation and IL-1β secretion in renal dendritic cells. Since the NLRP3 inflammasome activates pyroptosis, crystal-induced kidney injury may involve pyroptosis, although this possibility was not specifically addressed.

**Cell death in urinary tract obstruction**

Urinary tract obstruction is a frequent phenomenon in the clinic than is usually corrected by timely surgery or intervention. In addition, it has extensively been used as a preclinical model of accelerated chronic kidney disease ^38^. However, timely correction may not possible and a correct understanding of the molecular mechanisms leading to kidney atrophy may help preserve kidney health. Specifically, medical therapies may benefit neonatal ureteropelvic junction obstruction patients while the need for intervention is assessed ^39^. More than 30 years ago, apoptosis was identified by morphological criteria as the form of cell death associated with the loss of tubular cell mass occurring after urinary tract obstruction and leading to irreversible tubular atrophy and interstitial fibrosis ^40^. Both loss of tubular cell mass and apoptosis occurred from 1 week after permanent ureteric ligation, and were most rapid between 2 and 4 weeks. Inflammation and low levels of pro-survival factors are likely drivers of tubular cell apoptosis since deficiency of inflammatory cytokines or administration of pro-survival cytokines decreased tubular cell apoptosis ^41, 42^. Low levels of survival factors such as VEGF are also instrumental in the loss of peritubular capillaries driven by endothelial cell apoptosis ^43^. Classical intracellular mediators of apoptosis, including pro-apoptotic protein Omi/HtrA2 have been shown by *in vivo* functional studies to be involved ^44^. Most recently, dual deficiency of Bax and Bak inhibited tubular apoptosis and atrophy as well as subsequent inflammation and kidney fibrosis ^45^. In this regard, CD36-mediated phagocytosis of apoptotic cells may serve as an important pathway in the progression of fibrosis ^46^. However, despite the initial emphasis on the role of apoptosis, it is likely that diverse forms of cell death contribute to cell loss in different nephron segments: apoptosis and necrosis in proximal tubular cells and apoptosis in collecting ducts ^47^. In this regard, it is striking the paucity of reports on caspase targeting in urinary tract obstruction, despite evidence of caspase activation ^48^. Tubular cell-specific expression of the viral pan-caspase inhibitor p35 decreased inflammation and tubular cell apoptosis ^49^. However, it is unclear whether this effect was dependent on a primary effect on caspase-regulated inflammation or on cell death. Poly(ADP-ribose) polymerase 1 (PARP1) deficiency was also protective in this model. Although it improved tubular histology and limited inflammation and fibrosis, it did not modulate apoptosis, suggesting a role in necrosis ^50^. However, no reports were available in June 2017 on the presence or role of necroptosis, ferroptosis or regulated necrosis in triggering tubular cell death during urinary tract obstruction. Additionally, persistent autophagy in proximal tubules was proposed to be a driver of tubular cell death and interstitial fibrosis, based on the observation that these were suppressed by pharmacological inhibitors of autophagy as well as by interference with autophagy specifically in proximal tubules by a proximal tubule-specific knockout of autophagy-related 7 (PT-Atg7 KO) ^51^. Regarding myofibroblasts, which drive the fibrogenic response, therapeutic induction of myofibroblast apoptosis resulted in reduced interstitial fibrosis ^52^.

**HIF-dependent genes and pathway in renal cancer**

HIF-responsive genes such as VEGF, PDGF, and EGF have survival factor activity, while glucose transporters GLUT1 and GLUT4 favor the RCC reliance on aerobic glycolysis ^53^. The survival factor activity may increase RCC resistance to lethal stimuli and also increases tumor vascularization through several mechanisms, including increased survival of endothelial cells. In this regard, the current standard of RCC therapy involves anti-VEGF agents or tyrosine kinase inhibitors blocking VEGF receptor signaling ^2^. The HIF response also leads to p53 stabilization and increased survivin levels, as a consequence of the relocation of programmed cell death 5 (PDCD5) to the nucleus, where it leads to degradation of Mdm2 ^54^. HIF also increased the expression of the apoptosis inhibitor ARC (apoptosis repressor with a CARD: caspase activation and recruitment domain) ^55^. HIF2α stabilization makes VHL-deficient RCC cells resistant to natural killer (NK) cell-mediated killing by increasing the expression of ITPR1 (inositol 1,4,5-trisphosphaten receptor, type 1), that activates autophagy in response to NK-derived signals ^56^. Additional resistance of VHL-deficient RCC cells to mitochondria-activating apoptosis inducers results from increased expression of IGF1 receptors (IGF1R) which sensitize to the pro-survival activity of IGF1, resulting in AKT activation and Bak-Mcl-1 complex stabilization ^57^.

**REFERENCES**

1. Sanz AB, Justo P, Sanchez-Niño MD, Blanco-Colio LM, Winkles JA, Kreztler M*, et al.* The cytokine TWEAK modulates renal tubulointerstitial inflammation. *J Am Soc Nephrol* 2008, **19**(4)**:** 695-703.

2. Posadas EM, Limvorasak S, Figlin RA. Targeted therapies for renal cell carcinoma. *Nat Rev Nephrol* 2017, **13**(8)**:** 496-511.

3. Lobo N, Mount C, Omar K, Nair R, Thurairaja R, Khan MS. Landmarks in the treatment of muscle-invasive bladder cancer. *Nat Rev Urol* 2017.

4. Rijnders M, de Wit R, Boormans JL, Lolkema MPJ, van der Veldt AAM. Systematic Review of Immune Checkpoint Inhibition in Urological Cancers. *Eur Urol* 2017.

5. Kärkkäinen UM, Ikäheimo R, Katila ML, Siitonen A. Recurrence of urinary tract infections in adult patients with community-acquired pyelonephritis caused by E. coli: a 1-year follow-up. *Scand J Infect Dis* 2000, **32**(5)**:** 495-499.

6. Wang A, Nizran P, Malone MA, Riley T. Urinary tract infections. *Prim Care* 2013, **40**(3)**:** 687-706.

7. Mueller SP, Unger M, Guender L, Fekete A, Mueller MJ. Diacylglycerol acyltransferase-mediated triacylglyerol synthesis augments basal thermotolerance. *Plant Physiol* 2017.

8. Sanz AB, Santamaría B, Ruiz-Ortega M, Egido J, Ortiz A. Mechanisms of renal apoptosis in health and disease. *J Am Soc Nephrol* 2008, **19**(9)**:** 1634-1642.

9. Iurlaro R, Muñoz-Pinedo C. Cell death induced by endoplasmic reticulum stress. *FEBS J* 2016, **283**(14)**:** 2640-2652.

10. Mueller RB, Sheriff A, Gaipl US, Wesselborg S, Lauber K. Attraction of phagocytes by apoptotic cells is mediated by lysophosphatidylcholine. *Autoimmunity* 2007, **40**(4)**:** 342-344.

11. Elliott MR, Ravichandran KS. The Dynamics of Apoptotic Cell Clearance. *Dev Cell* 2016, **38**(2)**:** 147-160.

12. Schwab S, Jobin K, Kurts C. Urinary tract infection: recent insight into the evolutionary arms race between uropathogenic Escherichia coli and our immune system. *Nephrol Dial Transplant* 2017.

13. Tan CK, Carey AJ, Cui X, Webb RI, Ipe D, Crowley M*, et al.* Genome-wide mapping of cystitis due to Streptococcus agalactiae and Escherichia coli in mice identifies a unique bladder transcriptome that signifies pathogen-specific antimicrobial defense against urinary tract infection. *Infect Immun* 2012, **80**(9)**:** 3145-3160.

14. Zhang Z, Wang M, Eisel F, Tchatalbachev S, Chakraborty T, Meinhardt A*, et al.* Uropathogenic Escherichia coli Epigenetically Manipulate Host Cell Death Pathways. *J Infect Dis* 2016, **213**(7)**:** 1198-1207.

15. Walczak MJ, Puorger C, Glockshuber R, Wider G. Intramolecular donor strand complementation in the E. coli type 1 pilus subunit FimA explains the existence of FimA monomers as off-pathway products of pilus assembly that inhibit host cell apoptosis. *J Mol Biol* 2014, **426**(3)**:** 542-549.

16. Mulvey MA, Lopez-Boado YS, Wilson CL, Roth R, Parks WC, Heuser J*, et al.* Induction and evasion of host defenses by type 1-piliated uropathogenic Escherichia coli. *Science* 1998, **282**(5393)**:** 1494-1497.

17. Mysorekar IU, Mulvey MA, Hultgren SJ, Gordon JI. Molecular regulation of urothelial renewal and host defenses during infection with uropathogenic Escherichia coli. *J Biol Chem* 2002, **277**(9)**:** 7412-7419.

18. Thumbikat P, Berry RE, Zhou G, Billips BK, Yaggie RE, Zaichuk T*, et al.* Bacteria-induced uroplakin signaling mediates bladder response to infection. *PLoS Pathog* 2009, **5**(5)**:** e1000415.

19. Thumbikat P, Berry RE, Schaeffer AJ, Klumpp DJ. Differentiation-induced uroplakin III expression promotes urothelial cell death in response to uropathogenic E. coli. *Microbes Infect* 2009, **11**(1)**:** 57-65.

20. Klumpp DJ, Rycyk MT, Chen MC, Thumbikat P, Sengupta S, Schaeffer AJ. Uropathogenic Escherichia coli induces extrinsic and intrinsic cascades to initiate urothelial apoptosis. *Infect Immun* 2006, **74**(9)**:** 5106-5113.

21. Nagamatsu K, Hannan TJ, Guest RL, Kostakioti M, Hadjifrangiskou M, Binkley J*, et al.* Dysregulation of Escherichia coli α-hemolysin expression alters the course of acute and persistent urinary tract infection. *Proc Natl Acad Sci U S A* 2015, **112**(8)**:** E871-880.

22. Bauckman KA, Mysorekar IU. Ferritinophagy drives uropathogenic Escherichia coli persistence in bladder epithelial cells. *Autophagy* 2016, **12**(5)**:** 850-863.

23. Klumpp DJ, Weiser AC, Sengupta S, Forrestal SG, Batler RA, Schaeffer AJ. Uropathogenic Escherichia coli potentiates type 1 pilus-induced apoptosis by suppressing NF-kappaB. *Infect Immun* 2001, **69**(11)**:** 6689-6695.

24. Billips BK, Schaeffer AJ, Klumpp DJ. Molecular basis of uropathogenic Escherichia coli evasion of the innate immune response in the bladder. *Infect Immun* 2008, **76**(9)**:** 3891-3900.

25. Schaale K, Peters KM, Murthy AM, Fritzsche AK, Phan MD, Totsika M*, et al.* Strain- and host species-specific inflammasome activation, IL-1β release, and cell death in macrophages infected with uropathogenic Escherichia coli. *Mucosal Immunol* 2016, **9**(1)**:** 124-136.

26. Blomgran R, Zheng L, Stendahl O. Uropathogenic Escherichia coli triggers oxygen-dependent apoptosis in human neutrophils through the cooperative effect of type 1 fimbriae and lipopolysaccharide. *Infect Immun* 2004, **72**(8)**:** 4570-4578.

27. Gilbert NM, O'Brien VP, Lewis AL. Transient microbiota exposures activate dormant Escherichia coli infection in the bladder and drive severe outcomes of recurrent disease. *PLoS Pathog* 2017, **13**(3)**:** e1006238.

28. Chen M, Jahnukainen T, Bao W, Daré E, Ceccatelli S, Celsi G. Uropathogenic Escherichia coli toxins induce caspase-independent apoptosis in renal proximal tubular cells via ERK signaling. *Am J Nephrol* 2003, **23**(3)**:** 140-151.

29. Plotnikov EY, Morosanova MA, Pevzner IB, Zorova LD, Manskikh VN, Pulkova NV*, et al.* Protective effect of mitochondria-targeted antioxidants in an acute bacterial infection. *Proc Natl Acad Sci U S A* 2013, **110**(33)**:** E3100-3108.

30. Lu Y, Bhushan S, Tchatalbachev S, Marconi M, Bergmann M, Weidner W*, et al.* Necrosis is the dominant cell death pathway in uropathogenic Escherichia coli elicited epididymo-orchitis and is responsible for damage of rat testis. *PLoS One* 2013, **8**(1)**:** e52919.

31. Miller C, Kennington L, Cooney R, Kohjimoto Y, Cao LC, Honeyman T*, et al.* Oxalate toxicity in renal epithelial cells: characteristics of apoptosis and necrosis. *Toxicol Appl Pharmacol* 2000, **162**(2)**:** 132-141.

32. Sarica K, Yagci F, Bakir K, Erbagci A, Erturhan S, Uçak R. Renal tubular injury induced by hyperoxaluria: evaluation of apoptotic changes. *Urol Res* 2001, **29**(1)**:** 34-37.

33. Lu X, Gao B, Wang Y, Liu Z, Yasui T, Liu P*, et al.* Renal tubular epithelial cell injury, apoptosis and inflammation are involved in melamine-related kidney stone formation. *Urol Res* 2012, **40**(6)**:** 717-723.

34. Sun XY, Ouyang JM. New view in cell death mode: effect of crystal size in renal epithelial cells. *Cell Death Dis* 2015, **6:** e2013.

35. Mulay SR, Desai J, Kumar SV, Eberhard JN, Thomasova D, Romoli S*, et al.* Cytotoxicity of crystals involves RIPK3-MLKL-mediated necroptosis. *Nat Commun* 2016, **7:** 10274.

36. Meizner I, Bar-Ziv J. Prenatal ultrasonic diagnosis of short rib polydactyly syndrome, type I. A case report. *J Reprod Med* 1989, **34**(9)**:** 668-672.

37. Ludwig-Portugall I, Bartok E, Dhana E, Evers BD, Primiano MJ, Hall JP*, et al.* An NLRP3-specific inflammasome inhibitor attenuates crystal-induced kidney fibrosis in mice. *Kidney Int* 2016, **90**(3)**:** 525-539.

38. Ucero AC, Benito-Martin A, Izquierdo MC, Sanchez-Niño MD, Sanz AB, Ramos AM*, et al.* Unilateral ureteral obstruction: beyond obstruction. *Int Urol Nephrol* 2014, **46**(4)**:** 765-776.

39. Decramer S, Wittke S, Mischak H, Zürbig P, Walden M, Bouissou F*, et al.* Predicting the clinical outcome of congenital unilateral ureteropelvic junction obstruction in newborn by urinary proteome analysis. *Nat Med* 2006, **12**(4)**:** 398-400.

40. Gobe GC, Axelsen RA. Genesis of renal tubular atrophy in experimental hydronephrosis in the rat. Role of apoptosis. *Lab Invest* 1987, **56**(3)**:** 273-281.

41. Ucero AC, Benito-Martin A, Fuentes-Calvo I, Santamaria B, Blanco J, Lopez-Novoa JM*, et al.* TNF-related weak inducer of apoptosis (TWEAK) promotes kidney fibrosis and Ras-dependent proliferation of cultured renal fibroblast. *Biochim Biophys Acta* 2013.

42. Gao X, Mae H, Ayabe N, Takai T, Oshima K, Hattori M*, et al.* Hepatocyte growth factor gene therapy retards the progression of chronic obstructive nephropathy. *Kidney Int* 2002, **62**(4)**:** 1238-1248.

43. Ohashi R, Shimizu A, Masuda Y, Kitamura H, Ishizaki M, Sugisaki Y*, et al.* Peritubular capillary regression during the progression of experimental obstructive nephropathy. *J Am Soc Nephrol* 2002, **13**(7)**:** 1795-1805.

44. Docherty NG, O'Sullivan OE, Healy DA, Fitzpatrick JM, Watson RW. Evidence that inhibition of tubular cell apoptosis protects against renal damage and development of fibrosis following ureteric obstruction. *Am J Physiol Renal Physiol* 2006, **290**(1)**:** F4-13.

45. Jang HS, Padanilam BJ. Simultaneous deletion of Bax and Bak is required to prevent apoptosis and interstitial fibrosis in obstructive nephropathy. *Am J Physiol Renal Physiol* 2015, **309**(6)**:** F540-550.

46. Pennathur S, Pasichnyk K, Bahrami NM, Zeng L, Febbraio M, Yamaguchi I*, et al.* The macrophage phagocytic receptor CD36 promotes fibrogenic pathways on removal of apoptotic cells during chronic kidney injury. *Am J Pathol* 2015, **185**(8)**:** 2232-2245.

47. Forbes MS, Thornhill BA, Minor JJ, Gordon KA, Galarreta CI, Chevalier RL. Fight-or-flight: murine unilateral ureteral obstruction causes extensive proximal tubular degeneration, collecting duct dilatation, and minimal fibrosis. *Am J Physiol Renal Physiol* 2012, **303**(1)**:** F120-129.

48. Truong LD, Choi YJ, Tsao CC, Ayala G, Sheikh-Hamad D, Nassar G*, et al.* Renal cell apoptosis in chronic obstructive uropathy: the roles of caspases. *Kidney Int* 2001, **60**(3)**:** 924-934.

49. Inoue T, Kusano T, Tomori K, Nakamoto H, Suzuki H, Okada H. Effects of cell-type-specific expression of a pan-caspase inhibitor on renal fibrogenesis. *Clin Exp Nephrol* 2015, **19**(3)**:** 350-358.

50. Kim J, Padanilam BJ. Loss of poly(ADP-ribose) polymerase 1 attenuates renal fibrosis and inflammation during unilateral ureteral obstruction. *Am J Physiol Renal Physiol* 2011, **301**(2)**:** F450-459.

51. Livingston MJ, Ding HF, Huang S, Hill JA, Yin XM, Dong Z. Persistent activation of autophagy in kidney tubular cells promotes renal interstitial fibrosis during unilateral ureteral obstruction. *Autophagy* 2016, **12**(6)**:** 976-998.

52. Wang W, Zhou PH, Xu CG, Zhou XJ, Hu W, Zhang J. Baicalein ameliorates renal interstitial fibrosis by inducing myofibroblast apoptosis in vivo and in vitro. *BJU Int* 2016, **118**(1)**:** 145-152.

53. Ciccarese C, Brunelli M, Montironi R, Fiorentino M, Iacovelli R, Heng D*, et al.* The prospect of precision therapy for renal cell carcinoma. *Cancer Treat Rev* 2016, **49:** 37-44.

54. Essers PB, Klasson TD, Pereboom TC, Mans DA, Nicastro M, Boldt K*, et al.* The von Hippel-Lindau tumor suppressor regulates programmed cell death 5-mediated degradation of Mdm2. *Oncogene* 2015, **34**(6)**:** 771-779.

55. Razorenova OV, Castellini L, Colavitti R, Edgington LE, Nicolau M, Huang X*, et al.* The apoptosis repressor with a CARD domain (ARC) gene is a direct hypoxia-inducible factor 1 target gene and promotes survival and proliferation of VHL-deficient renal cancer cells. *Mol Cell Biol* 2014, **34**(4)**:** 739-751.

56. Messai Y, Noman MZ, Hasmim M, Janji B, Tittarelli A, Boutet M*, et al.* ITPR1 protects renal cancer cells against natural killer cells by inducing autophagy. *Cancer Res* 2014, **74**(23)**:** 6820-6832.

57. Yamaguchi R, Harada H, Hirota K. VHL-deficient renal cancer cells gain resistance to mitochondria-activating apoptosis inducers by activating AKT through the IGF1R-PI3K pathway. *Tumour Biol* 2016, **37**(10)**:** 13295-13306.
